# Enhancing the Interfacial Property Between UHMWPE Fibers and Epoxy Through Polydopamine and SiO_2_ Surface Modification

**DOI:** 10.1002/open.202400131

**Published:** 2024-10-25

**Authors:** Nengjun Ben, Sulun Jiang, Liping Zhao, Jiale Gong, Lu Shen, Kui Wang, Chenyang Tang

**Affiliations:** ^1^ Intelligent agricultural equipment collaborative innovation center of Ministry of Education Yancheng Polytechnic College South Jiefang Road No. 285 Yancheng 224051 PR China; ^2^ Institute of Chemical and Industrial Bioengineering Jilin Engineering Normal University Kaixuan Road No. 3050 Changchun 130052 PR China; ^3^ School of Materials Science and Engineering Yancheng Institute of Technology Hope Avenue Road No.1 Yancheng 224051 PR China

**Keywords:** Interfaces, Nanoparticles, Nanostructures, Polymers, Silicon

## Abstract

Here, a combination of dopamine self‐polymerization and epoxy modified SiO_2_ (M‐SiO_2_) grafting was proposed, with the purpose of increasing interfacial adhesion of UHMWPE fiber. Inspired by mussel adhesion, polydopamine (PDA) was deposited onto the surface of UHMWPE fiber to form a thin layer with amino and hydroxyl groups. M‐SiO_2_ nanoparticles were then adhered to fiber surface via chemical reactions by a “two‐step” or “one‐step” technology. In the “two‐step” technique, the M‐SiO_2_ nanoparticles were adhered to the surface of PDA modified UHMWPE fiber via reactions between epoxy and amino groups. In the “one‐step” method, M‐SiO_2_ and dopamine were added into the UHMWPE/Tris solution at the same time. Surface morphology and thermal properties of various UHMWPE fibers were tested by SEM and TGA, respectively. Surface wettability of different UHMWPE fibers were evaluated by dynamic contact angle. The results proved that PDA and M‐SiO_2_ were successfully adhered to the surface of UHMWPE fibers. The mechanical property of modified UHMWPE/Epoxy composites was investigated, and 43.7 % improvement was obtained, compared with unmodified UHMWPE/Epoxy composite. Additionally, micro‐bond test revealed that the interfacial property (IFSS value) of modified UHMWPE fiber via the “one‐step” method was 6.08 MPa, significantly higher than that of unmodified UHMWPE fiber (2.47 MPa).

## Graphical abstract

UHMWPE fiber with improved interfacial adhesion was fabricated by grafting modified SiO_2_ and PDA simultaneously onto the surface of UHMWPE fiber. After modification, the mechanical and interfacial properties of modified UHMWPE/Epoxy composites via the “one‐step” method were increased by 43.7 % and 146 %, respectively, compared to unmodified UHMWPE fiber.

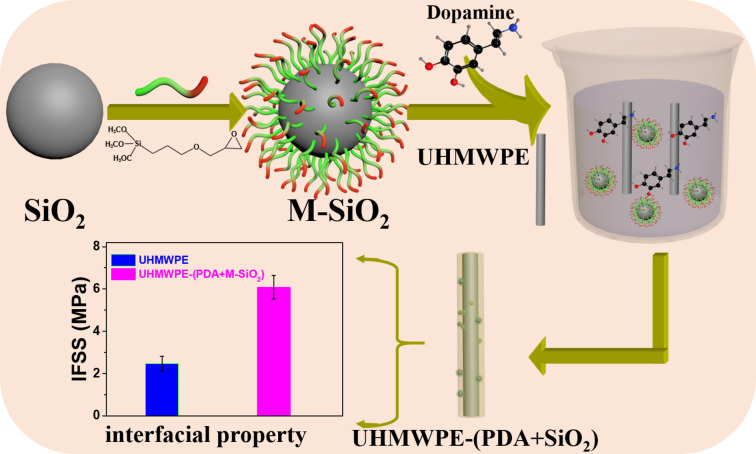

## 1 Introduction

Ultra‐high molecular weight polyethylene (UHMWPE) fiber is one kind of commonly used high performance fibers, which possesses high strength, high modulus and other excellent properties.^[1]^ Thus, UHMWPE fiber represents a bright prospect in multiple fields, for instance military, sports, construction areas et al. Unfortunately, the compatibility of UHMWPE fiber with resin is very poor, due to its high crystallinity, highly smooth surface, and lack of polar groups. Therefore, the fiber‐reinforced composite materials with the poor compatibility exhibit a shortened lifespan and an increased failure probability, restricting their widespread application in the aforementioned fields. To address this issue, the surface of UHMWPE fiber needs to be modified to generate polar groups on the surface, thereby enhancing surface polarity and improving the compatibility between fiber and the resin as well as the interfacial interactions.

The processing methods of UHMWPE fiber are mainly divided into blending method and surface treatment method.^[2]^ The blending method involves adding polar substances to the raw UHMWPE materials for subsequent spinning. Nevertheless, the compatibility between the polar substances and UHMWPE is weak, and the molecular weight of the polar substances is lower than that of UHMWPE. Because of this, it is challenging to prepare UHMWPE fiber with high orientation and crystallization, which is detrimental to producing fiber products with excellent properties. Consequently, surface treatment method has emerged as the most popular way for producing UHMWPE fibers with good adhesion, which involves adhering micro‐molecule, polymer or nanoparticle onto the fiber surface via chemical bonding or physical treatment method.^[3]^ Conventional surface treatment techniques include oxidation treatment,^[4]^ plasma treatment,^[5]^ irradiation treatment,^[6]^ and surface grafting method,^[7]^ et al. Nevertheless, the aforementioned approaches still have drawback, as traditional methods adhere polar groups onto UHMWPE fiber through disrupting its high crystalline and oriented structure, whereas the orderly structure of UHMWPE is the reason for its excellent mechanical properties. Therefore, conventional surface treatment may result in a partial decrease in mechanical properties, while it can increase interfacial adhesion. In addition, harsh and expensive reaction conditions are pressing problems that conventional surface treatment techniques must solve.

To address the issues of structure damage and decreased mechanical properties, polar groups should adhere to the fiber surface through physical interactions. However, few substances meet this requirement. In recent years, dopamine, an extract of mussels, can firmly adhere to the surface of various materials through hydrogen bonding, van der Waals forces, et al.^[8]^ Moreover, dopamine coating conditions are mild, causing no damage to the structure of the substrate material. Therefore, dopamine is frequently employed as an adhesive for fiber surface modification.^[9]^ Ming Feng deposited a copper‐polydopamine (PDA) complex coating on the surface of UHMWPE fibers through chemical copper plating and dopamine self‐polymerization.^[10]^ In comparison to the pure rigid polyurethane (RPU) composite, the PDA−Cu‐UHMWPE/RPU composite exhibited increases in interlaminar shear strength, tensile strength, and impact strength of 35.85 %, 97.8 %, and 2456.9 %, respectively. Suman Chhetri prepared a stable double‐layer coating on the surface of UHMWPE fiber by utilizing the super adhesion of dopamine.^[11]^ Firstly, PDA layer was coated on the surface through physical interactions, and then Polyamide 6,6 could adhere to the surface of PDA layer via hydrogen bonding. Compared with untreated UHMWPE fiber, the tensile strength of PDA‐Polyamide 6,6‐UHMWPE fiber increased by 11 %, In addition, the interfacial shear strength (IFSS) of PDA‐Polyamide 6,6‐UHMWPE fiber/epoxy composite increased by 40 % compared to untreated UHMWPE fiber.

In this study, UHMWPE fiber with improved adhesion was fabricated by grafting modified SiO_2_ and PDA onto the surface of UHMWPE fiber. Firstly, SiO_2_ was modified using a silane coupling agent to obtain the modified SiO_2_ covered with epoxy groups. The successful modification of SiO_2_ was confirmed by SEM, XPS and TGA tests. Then, two methods were employed to produce SiO_2_‐modified UHMWPE fiber: a) epoxy modified SiO_2_ (M‐SiO_2_) was grafted onto PDA modified UHMWPE fiber (UHMWPE‐PDA) surface, utilizing chemical reactions between epoxy of M‐SiO_2_ and amino groups of PDA. While PDA could firmly adhere to UHMWPE fiber surface through hydrogen bonding and van der Waals forces. b) both M‐SiO_2_ and dopamine were added into the solution simultaneously. Then a compound coating on the fiber surface was formed on UHMWPE fiber surface via the adhesion of dopamine, as well as the reactions between epoxy groups of M‐SiO_2_ and amino groups of dopamine. PDA and M‐SiO_2_ were successfully coated on the fiber surface, as proved by SEM and TGA tests. Subsequently, different tests were chosen to assess the performance of the two kinds modified UHMWPE fiber. Dynamic contact angle tests revealed that the contact angle of the two types modified UHMWPE fiber were significantly reduced, while the second method resulting in a low value. Additionally, the properties of the two modified UHMWPE fibers were investigated, when they were utilized as the reinforcing phase to create fiber‐reinforced composites. The outcomes showed that both the mechanical properties and interfacial performance of modified UHMWPE/Epoxy composites were improved, with the second technique producing a higher enhancement.

## 2 Results and Discussion

### 2.1 Characterizations of SiO_2_ and M‐SiO_2_.

**Figure 1:**
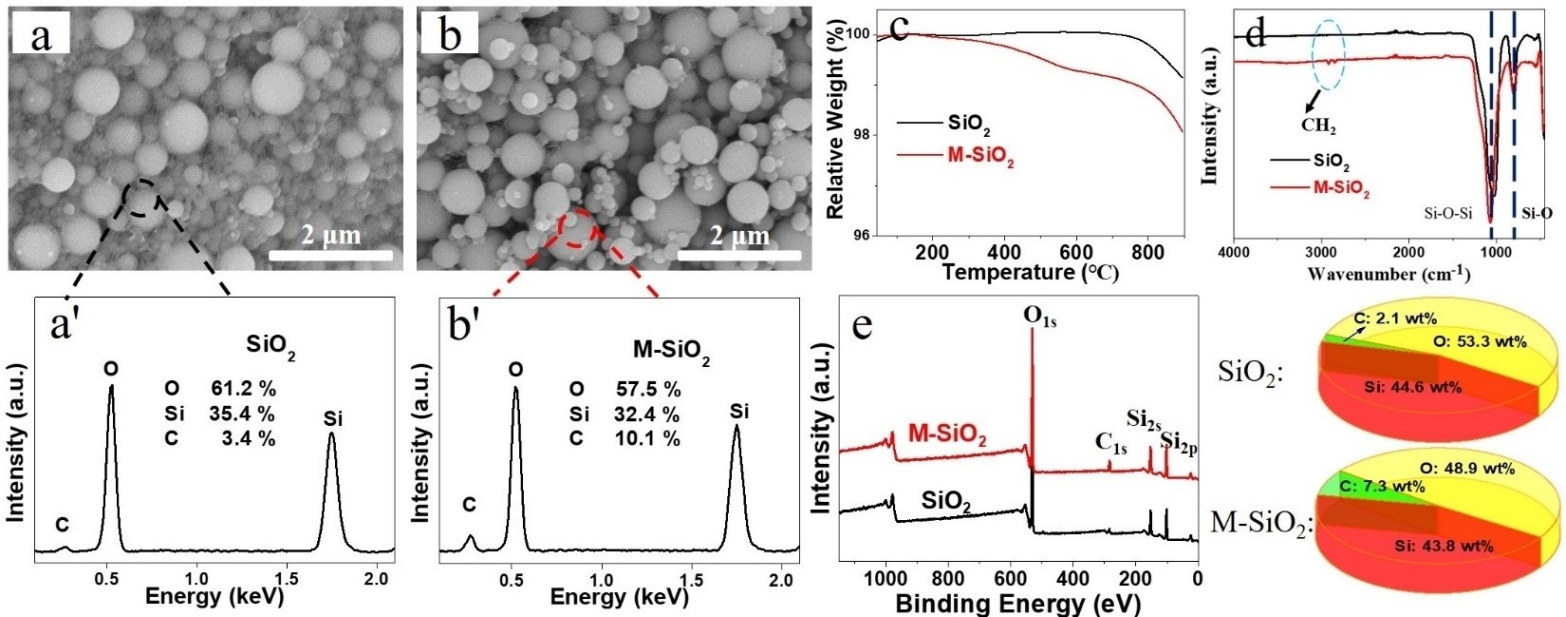
Microstructure and EDS results (wt %) of SiO_2_ (a, a′) and M‐SiO_2_ (b, b′), TGA (c), FTIR (d) and XPS (e) spectra of SiO_2_ before and after modification.

The micromorphology of SiO_2_ before and after modification was depicted in Figures 1a–b. It could be observed from the figures that SiO_2_ materials were spherical nanoparticles before modification, and the spherical structure was maintained after modification. The reason of this phenomenon was that the reaction condition between hydroxyl groups of SiO_2_ and silane coupling agent was moderate, without structure damage of SiO_2_. Furthermore, EDS test was also used to detect surface element contents of different SiO_2_, as shown in Figures 1a′ and b′. For the unmodified SiO_2_, the O and Si element contents were 61.2 and 35.4 wt %, respectively. After GPTES modification, those contents of M‐SiO_2_ decreased to 57.5 and 32.4 wt %, respectively, while the C element content increased significantly, rising from 3.4 wt % to 10.1 wt %. As illustrated in TGA curves (Figure 1c), the weight of unmodified SiO_2_ remained relatively stable, while partial decomposition occurred around 700 °C. At 900 °C, the value of residue was roughly 99.1 %. Regarding M‐SiO_2_, thermal degradation started at 300 °C., the weight contents were 99.1 wt % and 98.0 wt % at 700 °C and 900 °C, respectively. There was no doubt that the change in thermal decomposition temperature was due to new material adsorbed onto the surface of M‐SiO_2_, which confirmed the attachment of silane coupling agent GPTES.

**Scheme 1:**
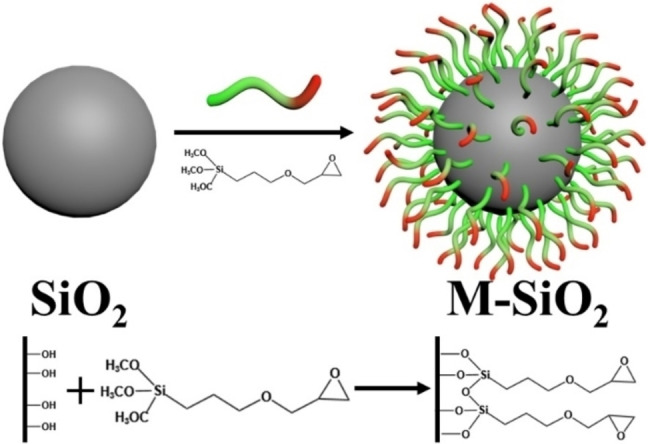
The fabrication schematic of M‐SiO_2_.

The variations in functional groups and element contents on the surface of different SiO_2_ were shown in Figures 1d–e, respectively. The two distinctive absorption peaks, which correspond to the Si−O−Si antisymmetric stretching vibration and the Si−O symmetric stretching vibration, were evident in the FTIR spectra (Figure 1d) both before and after modification. Furthermore, the freshly formed M‐SiO_2_ peaks at 2918 and 2847 cm^−1^ were the methylene peaks. Combined with the structure of GPTES, it could be concluded that the material coated on the surface of M‐SiO_2_ was GPTES.^[12]^ Additionally, the element contents on the surface of different SiO_2_ samples were examined by XPS. According to the result, there was a small amount of C element (2.1 wt %) on the surface of SiO_2_ before modification. This could have been caused by an impurity during the production process. Following GPTES adherence, the contents of Si and O decreased while the C element content increased dramatically, rising from 2.1 wt % to 7.3 wt %. The variations of the three element contents in XPS measurement was consistent with that of EDS test, further proving the grafting of GPTES on the surface of M‐SiO_2_. Hence, the preparation process of M‐SiO_2_ could be inferred, and the mechanism was illustrated in Scheme 1.

### 2.2 Effectiveness of M‐SiO_2_ Coated on UHMWPE in Different Methods

**Figure 2:**
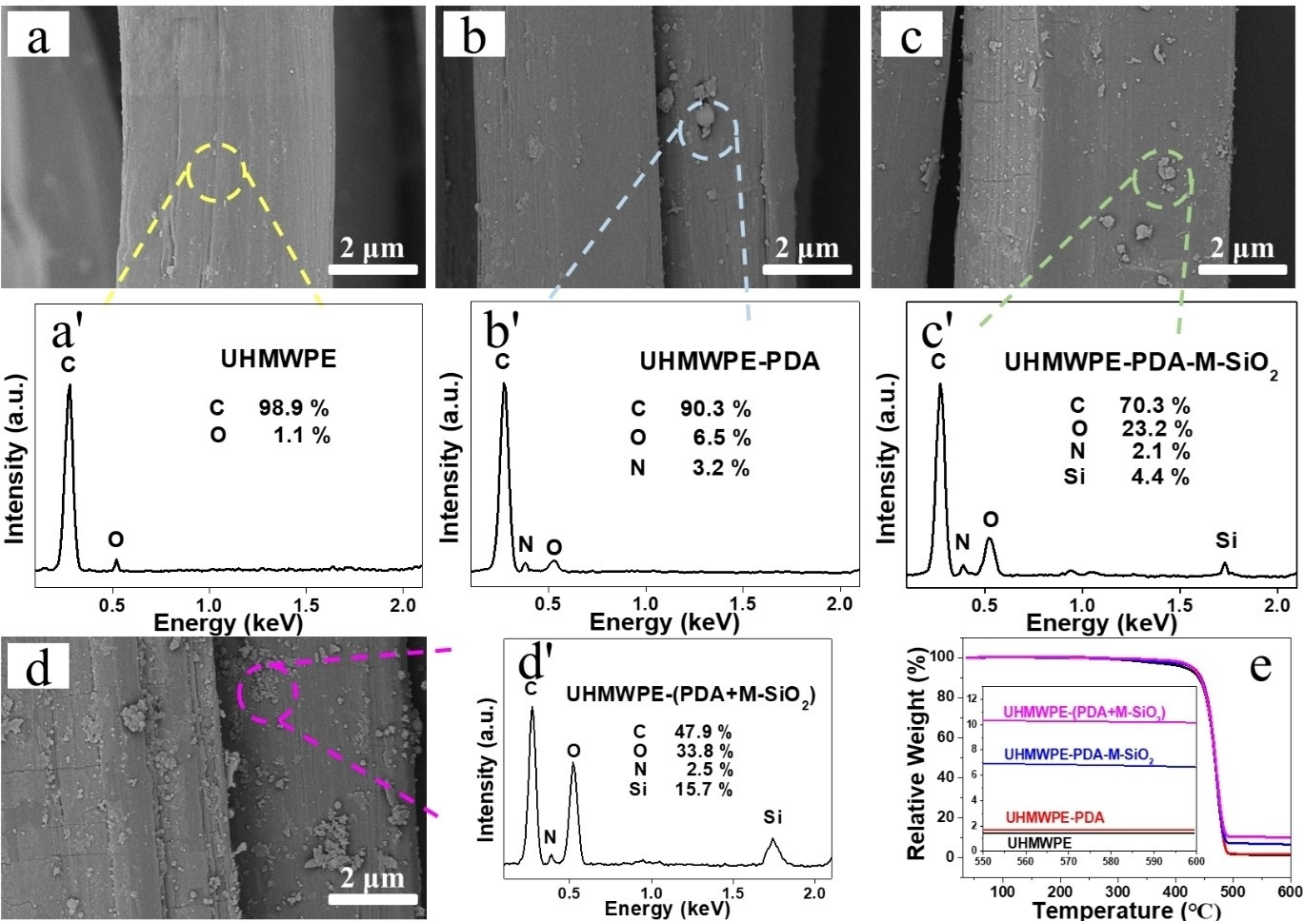
Surface morphology and EDS (wt %) of UHMWPE (a, a′), UHMWPE‐PDA (b, b′), UHMWPE‐PDA‐M‐SiO_2_ (c, c′) and UHMWPE‐(PDA+M‐SiO_2_) (d, d′), TGA (e) spectra of different UHMWPE fibers.

Surface morphology and thermal properties of various UHMWPE fibers were illustrated in Figure 2. Figure 2a showed that the unmodified UHMWPE fiber had a smooth surface with a few grooves aligned along the axis, and the grooves were created during the manufacture process. The content of C element on the UHMWPE surface was 98.9 %, which was significantly higher than the content of O element (1.1 %), according to elemental analysis. After PDA modified UHMWPE fiber, some spots emerged on the surface of UHMWPE‐PDA fiber. Combined with the EDS test (Figures 2b–b′), new emergent N element and increased O element content further proved successful coating of PDA on UHMWPE‐PDA surface. For UHMWPE‐PDA‐M‐SiO_2_, the surface became rougher after M‐SiO_2_ modification in Figure 2c. Testing results of the rough area indicated that the content of N element decreased (from 3.2 to 2.1 %), as well as newly emerged Si element. This variation was attributed to the shield of PDA with M‐SiO_2_, leading to a low content of N element. For another modification method, UHMWPE‐(PDA+M‐SiO_2_) in Figure 2d, a large amount of nanoparticles were observed on the surface. Obviously, these nanoparticles were SiO_2_. In addition, EDS measurement results demonstrated a notable increase in Si content (up to 15.7 %). Meanwhile, increased O content and reduced C content in Figure 2d′ also verified successful coating of PDA and M‐SiO_2_. Moreover, thermal properties of UHMWPE fibers before and after modification were also studied (Figure 2e). The values of residue content gradually increased from unmodified UHMWPE (1.41 %) to the two kinds of M‐SiO_2_ modified UHMWPE fibers (up to 6.71 % and 10.15 %). The increasing residue content indirectly indicated that M‐SiO_2_ and PDA had been coated on the surface of modified UHMWPE fibers. The more M‐SiO_2_ adhered to fiber surface, the rougher the surface became, which was consistent with SEM results.

### 2.3 Wettability of Different Fibers

**Scheme 2:**
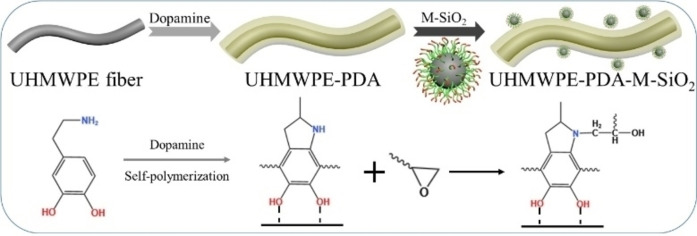
The preparation schematic of UHMWPE‐PDA‐M‐SiO_2_.

Surface wettability of UHMWPE fibers before and after modification were also studied. Corresponding dynamic contact angle and surface energy ($\gamma_{PE}$ , $\gamma_{PE}^{p}$ and $\gamma_{PE}^{d}$ ) were shown in Table 1. It could be seen from this table that the contact angle of unmodified UHMWPE was the highest, with the values of 102.4° (H_2_O) and 73.4° (CH_2_I_2_). After PDA and M‐SiO_2_ attachment, the contact angle of modified UHMWPE fibers reduced, attributing to modification increasing polar groups content on fiber surface. For UHMWPE‐PDA, the contact angle between the fiber and H_2_O decreased significantly to 88.3°, and the contact angle with CH_2_I_2_ also showed a similar trend, down to 69.7°. The contact angle of UHMWPE‐PDA−M‐SiO_2_, which obtained via the first method in Scheme 2, further decreased, with values of 85.7° and 66.2° for H_2_O and CH_2_I_2_, respectively. This variation was entirely predictable, due to the abundant epoxy groups on the surface of M‐SiO_2_. Compared with amino groups, epoxy groups illustrated better wettability with H_2_O. For UHMWPE‐(PDA+M‐SiO_2_), the values of contact angle with H_2_O and CH_2_I_2_ were the lowest, dropping down to 80.6° and 61.4°, respectively. There were two reasons for this phenomenon. Firstly, more oxygen‐containing functional groups existed on UHMWPE‐(PDA+M‐SiO_2_) fiber surface compared with UHMWPE‐PDA‐M‐SiO_2_, which could be observed from EDS results in Figure 2d′. Secondly, UHMWPE‐(PDA+M‐SiO_2_) fiber surface became more uneven than UHMWPE‐PDA‐M‐SiO_2_ (Figures 2c–d). It was well known that rough surface was conducive to improving wettability in the same condition. Hence UHMWPE‐(PDA+M‐SiO_2_) demonstrated a lower contact angle, compared with UHMWPE‐PDA‐M‐SiO_2_.

**Table 1:** Different values of contact angles and surface energy for various UHMWPE fibers.

	contact angles (°)		$\gamma_{PE}$ (mN/m)	$\gamma_{PE}^{p}$ (mN/m)	$\gamma_{PE}^{d}$ (mN/m)
	H_2_O	CH_2_I_2_			
UHMWPE	102.4±5.3	74.2±3.9	20.59	1.28	19.31
UHMWPE‐PDA	88.3±4.1	69.7±4.3	24.96	5.64	19.32
UHMWPE‐PDA‐M‐SiO_2_	85.7±3.6	66.2±4.1	27.14	6.18	20.95
UHMWPE‐(PDA+M‐SiO_2_)	80.6±4.5	61.4±3.4	30.73	7.85	22.88

**Scheme 3:**
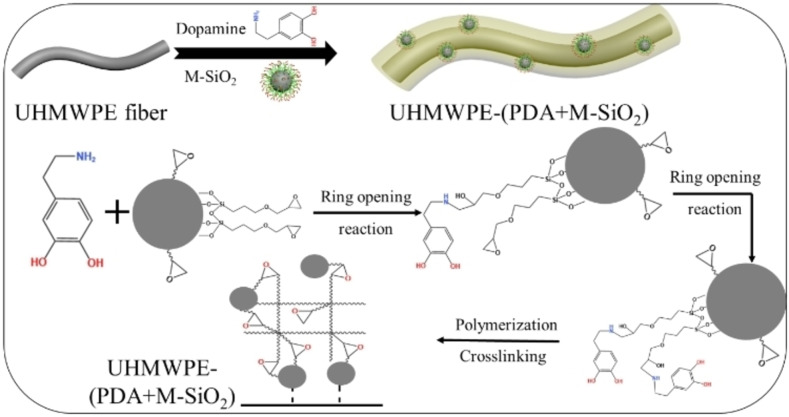
The fabrication schematic of UHMWPE‐(PDA+M‐SiO_2_).

In addition, the wettability of fiber could also be reflected by the value of surface energy. Table 1 displayed surface energy of various UHMWPE fibers. With a surface energy of just 20.59 mN/m, the unmodified UHMWPE fiber possessed the lowest surface energy among the four types of fibers. After modification, the surface energy gradually increased. The surface energy of UHMWPE‐(PDA+M‐SiO_2_) was the highest, with the value of 30.73 mN/m. This result was in consistent with the change in contact angle. That was, the smaller the contact angle was, the higher the surface energy became. This indicated that the fibers with the best wettability were prepared by the second method. Furthermore, the preparation schematic of UHMWPE‐(PDA+M‐SiO_2_) was shown in Scheme 3.

### 2.4 Mechanical Property

Mechanical properties of different UHMWPE fiber‐reinforced epoxy composites were also studied, and the results were shown in Table 2. According to Table 2, the tensile strength of UHMWPE fiber before modification was only 88.6 MPa, and the tensile modulus was 3.18 GPa. For UHMWPE‐PDA/Epoxy, the tensile strength was 102.8 MPa, which was 116 % higher than that of UHMWPE/Epoxy. This was due to the introduction of hydroxyl and amino groups onto the fiber surface, resulting in an increasing surface energy and wettability of the fiber. The tensile strength of UHMWPE‐PDA−M‐SiO_2_/Epoxy increased by 33.9 % to 118.6 MPa. The reason was that the surface of M‐SiO_2_ was covered with abundant epoxy groups, exhibiting a higher compatibility with epoxy resin than UHMWPE‐PDA. Moreover, the presence of M‐SiO_2_ on UHMWPE‐PDA‐M‐SiO_2_ surface would increase surface roughness. For UHMWPE‐(PDA+M‐SiO_2_), the fiber‐reinforced epoxy composite demonstrated the highest tensile strength (127.3 MPa). This was because during the preparation, PDA and M‐SiO_2_ were added into solution simultaneously, then PDA and M‐SiO_2_ could form a compound coating on the fiber surface through different interactions. For instance, the epoxy groups of M‐SiO_2_ could not only react with amino groups of PDA to establish chemical bonding, but also form hydrogen bonding with fiber surface, thereby enhancing the interactions between the compound coating and fiber surface. More significantly, M‐SiO_2_ could also act as a cross‐linking point in the compound coating, facilitating stress to be evenly and rapidly transferred from epoxy matrix to UHMWPE fibers.

**Table 2:** Mechanical properties of different fiber/Epoxy composites.

Sample	Tensile strength (MPa)	Tensile strength retention (%)	Tensile modulus (GPa)	Strain at break (%)
UHMWPE/Epoxy	88.6±8.9	100	3.18±0.24	8.5±1.4
UHMWPE‐PDA/Epoxy	102.8±7.3	116.0	3.23±0.28	7.8±1.2
UHMWPE‐PDA‐M‐SiO_2_/Epoxy	118.6±8.6	133.9	3.29±0.36	8.9±1.5
UHMWPE‐(PDA+M‐SiO_2_)/Epoxy	127.3±9.2	143.7	3.51±0.27	9.1±1.7

### 2.5 Interfacial Properties of Various UHMWPE Fiber‐Reinforced Epoxy Composites

**Figure 3:**
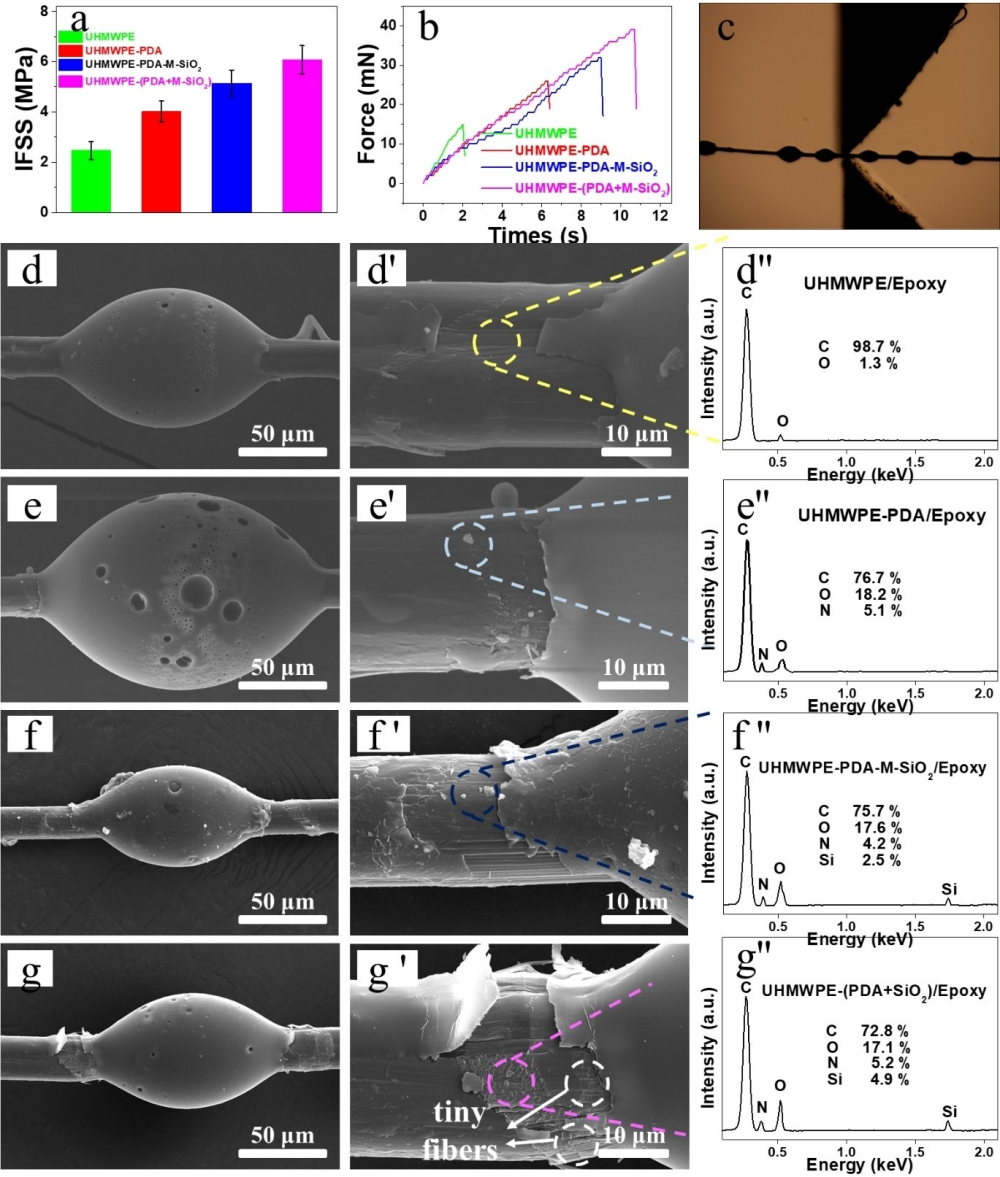
Average IFSS results (a), corresponding UHMWPE fiber/epoxy composites force–time curves (b), micro‐bond test picture (c), microstructure and EDS measurement (wt %) of UHMWPE/Epoxy (d–d′′), UHMWPE‐PDA/Epoxy (e–e′′), UHMWPE‐PDA‐M‐SiO_2_/Epoxy (f–f′′), and UHMWPE‐(PDA+M‐SiO_2_)/Epoxy (g–g′′)

Interfacial property between the fiber and epoxy matrix was the most significant factor influencing the performance of fiber‐reinforced epoxy composites. Therefore, interfacial properties of various UHMWPE/Epoxy composites were evaluated through micro‐bond test. Figure 3a illustrated IFSS values of different UHMWPE/Epoxy composites, and the values for each type UHMWPE/Epoxy composite were averaged over 30 testing outcomes. The curves in Figure 3b were force–time curves, which displaying the force changed as time went on. The force increased over time, therefore there was no direct correlation between the slope of these curves and the interfacial performance of UHMWPE/Epoxy composites.

As can be seen from Figure 3a, the IFSS value of UHMWPE/Epoxy before modification was the lowest, only 2.47 MPa. This result was reasonable, because the surface of UHMWPE fiber was smooth and there was no polar groups, resulting in weak interactions between UHMWPE fiber and epoxy matrix. As a result, only a small force (15 mN) was required to pull fiber out of epoxy micro‐droplet. For UHMWPE‐PDA, the IFSS increased to 4.02 MPa, attributing to the formation of hydrogen bonding and chemical bonding between PDA and epoxy matrix. As to UHMWPE‐PDA‐M‐SiO_2_, its interfacial performance was further improved, and the IFSS was 5.14 MPa. According to SEM results in Figure 2c, UHMWPE‐PDA‐M‐SiO_2_ fiber surface was grafted with M‐SiO_2_ after modification. Because of epoxy groups and uneven diameter distribution of M‐SiO_2_ on fiber surface, the polarity and roughness of UHMWPE‐PDA‐M‐SiO_2_ increased, facilitating interactions between the fiber and epoxy matrix. The fiber/epoxy composite with the best interfacial performance was UHMWPE‐(PDA+M‐SiO_2_), which increased by 146 % from 2.47 MPa to 6.08 MPa. The reasons of this IFSS improvement were illustrated as follows: i) Both M‐SiO_2_ and PDA were coated simultaneously, then a compound coating on the fiber surface was formed on UHMWPE fiber surface. There were many polar groups in this compound layer, which could form different interactions with epoxy matrix, such as hydrogen bonding, van der Waals force or chemical bonding. ii) The surface of M‐SiO_2_ existed a large number of epoxy groups, which might serve as an anchor in the compound coating. By strengthening mechanical interlocking, these anchors in the layer securely bonded M‐SiO_2_ and PDA together. iii) Furthermore, because M‐SiO_2_ was present in the compound coating, it may also serve as a crosslinking point, allowing the force to transmit evenly and quickly from epoxy matrix to UHMWPE fiber. iv) Covalent bonding between M‐SiO2 and PDA helped to create a continuous crack path and reduce the concentration of stress.

Surface morphology of various fiber/epoxy micro‐droplets after micro‐bond testing were also studied, and the results were displayed in Figures 3d–g′′. Figure 3d showed that no obvious small particles were observed on the surface of unmodified UHMWPE fiber after the micro‐bond testing. This was because the surface of UHMWPE was nonpolar, highly crystalline and extremely smooth. This phenomenon was verified by EDS testing in Figure 3d′, only detecting C element (98.7 %) and a small amount of oxygen element (1.3 %). The little amount of oxygen element was produced during UHMWPE fiber production process, which was also found in unmodified UHMWPE (Figures 2a–a′). For UHMWPE‐PDA, a small particle was presented in the debonding area in Figure 3e′, and EDS testing indicated that the particle should be epoxy resin remaining on the fiber surface. In contrast to UHMWPE‐PDA, the debonding area of UHMWPE‐PDA‐M‐SiO_2_ became rougher, and more particles were observed. Based on EDS masurement in Figure 3f′, Si element was introduced onto fiber surface, and the content was 2.5 %. Apparently, these particles were the mixture of PDA, M‐SiO_2_ and epoxy resin, suggesting strong interactions between fiber and epoxy resin. Regarding UHMWPE‐(PDA+M‐SiO_2_), continuous small particles were found in the debonding area, and element analysis revealed that Si element content was 4.9 %. When the debonding area in Figure 3g′ was magnified, plenty of tiny fibers were observed, which was not found in the debonding areas of other fibers. These tiny fibers should be monofilament, which broke apart from UHMWPE fibers under a larger force. This occurrence indirectly indicated that the interfacial interactions between UHMWPE‐(PDA+M‐SiO_2_) and epoxy matrix was the strongest among the four fiber/epoxy composites.

### 2.6 Interfacial Failure Mechanism

**Figure 4:**
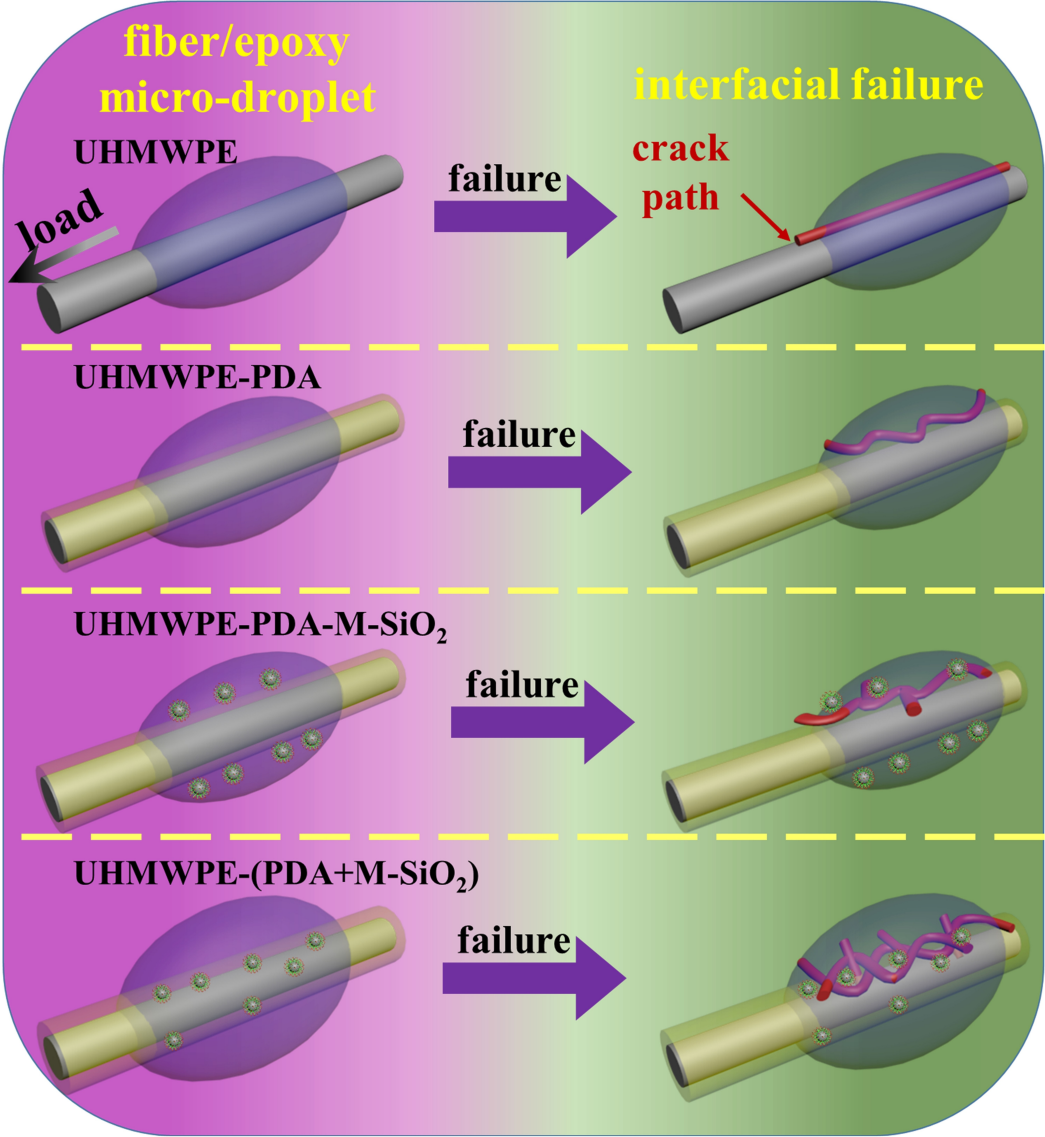
Interfacial failure mechanism of different UHMWPE fiber‐reinforced epoxy composites.

Interfacial failure mechanisms of various UHMWPE fiber/epoxy composites were also investigated, and relevant schematic diagrams were displayed in Figure 4. Because of the smooth and nonpolar surface, the force would be transmitted along the interface between UHMWPE fiber and epoxy matrix, when the UHMWPE/Epoxy composite subjected to external force, as seen in Figure 4. It was difficult to transfer the force from the epoxy matrix to the UHMWPE fiber surface, since the crack route would be straight at this interface. As a result, the crack path would be straight at this interface, therefore the force was difficult to be transmitted from epoxy matrix to UHMWPE fiber surface. In consequence, stress concentration and interface failure could occur under a small force, yielding the lowest IFSS value (2.47 MPa), and the interfacial failure mode was adhesive failure. Regarding UHMWPE‐PDA, amino and hydroxyl groups were generated on fiber surface after PDA grafted, leading to a notable rise in surface energy (from 20.59 to 24.96 mN/m). Because of the improved interactions between UHMWPE‐PDA and epoxy resin, the force could be effectively transferred from the epoxy matrix to the fiber. Moreover, the crack path became deflecting and complex, helping to achieve a high IFSS (4.02 MPa), and the failure mode was cohesive and substrate failure. In the case of UHMWPE‐PDA‐M‐SiO_2_, a large number of M‐SiO_2_ nanoparticles were adhered to the outside surface of PDA layer, and there contained epoxy groups on M‐SiO_2_ surface. Consequently, these epoxy groups guaranteed good compatibility between UHMWPE‐PDA‐M‐SiO_2_ and epoxy resin. When subjected to external force, the inorganic material M‐SiO_2_ might potentially improve the efficiency of stress transfer, resulting in a more complex crack path and a stronger interfacial interactions compared with UHMWPE‐PDA (5.14 MPa). Furthermore, Si element was still observed in the debonding region in Figure 3f′′, showing that M‐SiO_2_ was still present on UHMWPE‐PDA‐M‐SiO_2_ surface even after the fiber and epoxy matrix separated. Therefore, we could conclude that the failure mechanism was cohesive and substrate failure. As to UHMWPE‐(PDA+M‐SiO_2_), the interactions and IFSS were the strongest due to the following reasons: i) The compound coating, which formed through interactions between M‐SiO_2_ and PDA, possessed abundant polar groups. Thus different interactions could occur between epoxy matrix and this compound coating. ii) The abundance of epoxy groups on the surface of M‐SiO_2_ served as anchors for the compound layer. Afterwards, these anchors in the coating could strengthen mechanical interlocking, thus establishing a strong connection between M‐SiO_2_ and PDA. iii) Moreover, M‐SiO_2_, internal ingredients of this compound coating, also could act as a crosslinking point, allowing the force to transmit evenly and quickly from epoxy matrix to fiber. iv) The uniform distribution of M‐SiO_2_ in this compound coating and chemical bonding interconnections also caused a long and complicated crack path, significantly reducing stress concentration. As the force gradually increased, the compound coating separated from epoxy resin, leaving a small amount of coating. This result demonstrated that the force between the coating and the fiber was higher than that between the coating and epoxy resin. Here the failure mechanism was cohesive and substrate failure.

## 3 Conclusions

Modified UHMWPE fibers with a firm, uniformly distributed, and polar compound coating were successfully prepared via dopamine self‐polymerization and M‐SiO_2_ grafting. The preparation technology was eco‐friendly, simple and fast. The PDA and M‐SiO_2_ compound layer via the “one‐step” method possessed abundant polar groups, which could form strong interactions between fiber surface and epoxy matrix. Moreover, a large number of epoxy groups were existed on the surface of M‐SiO_2_ in this compound layer. Hence the M‐SiO_2_ not only served as an anchor in the compound coating via chemical reacted with PDA, but also acted as a crosslinking point to allow the force to transmit evenly and quickly from epoxy matrix to UHMWPE fiber. Compared to unmodified UHMWPE fiber, the as‐prepared PDA and M‐SiO_2_ modified UHMWPE fibers by the “one‐step” method exhibited 43.7 % and 146 % enhancement in the mechanical and interfacial properties, respectively. This study provided an efficient and universal method to prepare fibers with excellent performance.

## Experimental Section

### Materials

The diameter of UHMWPE fiber was 20 μm, and each bundle contains about 200 monofilament. The fiber was obtained from Hunan Zhongtai Special Equipment Co., Ltd., China. The average diameter of SiO_2_ was 500 μm, with the aim of improving interfacial performance. Tris (hydroxide) aminomethane, ethanol, and hydrochloric acid were acquired from Sinopharm Chemical Reagent Co., Ltd., China, dopamine and silane coupling agent 3‐Glycidyloxypropyltrimethoxysilane purchased from Aladdin Reagent Co., Ltd., China. Curing agent (593) and Epoxy resin (E 51) were obtained from Shenzhen Hengda Technology Trading Co., Ltd., China. Furthermore, all the reactants in this work were used as received.

### Sample Preparation

#### Preparation of Epoxy Modified SiO_2_ (M‐SiO_2_)

Firstly, a certain quantity of SiO_2_ powder was added into ethanol solution and sonicated for 30 minutes to guarantee uniform dispersion. In order to partially hydrolyze 3‐Glycidyloxypropyltrimethoxysilane (GPTES), a tiny amount of water and GPTES were added to the ethanol solution concurrently, and the mixture was stirred for 10 minutes. Subsequently, the well‐dispersed SiO_2_/H_2_O solution was then combined with the partially hydrolyzed GPTES/H_2_O solution. The mixture was centrifuged to extract the supernatant after of reacting 8 h at 60 °C, producing coarse product. After that, the coarse product was washed and centrifuged three times using a 1 : 1 ethanol/deionized water solution. After freeze‐drying, the epoxy‐modified SiO_2_ was obtained, which was abbreviated as M‐SiO_2_.

#### Preparation of M‐SiO_2_ Modified UHMWPE Fiber

The preparation process of PDA modified UHMWPE fiber (UHMWPE‐PDA) was reported in our previous work.^[9c]^

Adding a little amount of M‐SiO_2_ into 100 ml ethanol, and then sonicating for 0.5 h. After that, UHMWPE‐PDA fiber was immersed into the mixed M‐SiO_2_ solution for 8 h at 60 °C. At last, SiO_2_ modified UHMWPE fiber was obtained, and the fiber was identified as UHMWPE‐PDA‐M‐SiO_2_.

#### Preparation of M‐SiO_2_ and PDA Synergistically Modified UHMWPE Fiber

First, a certain amount of Tris was added into a beaker and stirred until it was completely dissolved. Next, the pH of the solution was adjusted to 8.5 with HCl. After stirring for a few minutes, add dopamine and M‐SiO_2_ into the Tris buffer solution. Five minutes later, the cleaned UHMWPE fiber was immersed into the mixed M‐SiO_2_/dopamine/Tris solution for 8 h at 60 °C. At last, M‐SiO_2_ and PDA synergistically modified UHMWPE fiber, marked as UHMWPE‐(PDA+M‐SiO_2_), was prepared.

#### Characterization

Surface morphology of SiO_2_ before and after modification, various UHMWPE fibers were assessed by Scanning electron microscope (Nova Nano SEM 450). The distribution of functional groups in various SiO_2_ was identified using Fourier transform infrared spectroscopy (FTIR, Nicolet 6700). Thermal property under argon of different SiO_2_ and various UHMWPE fibers were analyzed via a diamond TG/DTA Perkin Elmer instrument (TGA) at 10 °C/min. Surface element contents of different SiO_2_ were detected by an X‐ray photoelectron spectroscopy (XPS, Kratos AXIS). Interfacial property of various UHMWPE/Epoxy composites were assessed via a composite material interfacial evaluation equipment (MH410). Dynamic contact angle of various UHMWPE fibers was tested by a dynamic contact Angle analyzer (DCAT21). Tensile property of various UHMWPE/Epoxy composites were measured by a universal testing machine (Instron 5567) according to ASTM D 638.

## Supporting Information Summary

Additional supporting information can be found online in the Supporting Information section at the end of this article.

## Conflict of Interests

The authors declare no conflict of interest.

4

## Data Availability

The data that support the findings of this study are available from the corresponding author upon reasonable request.

## References

[1] (a) W. Li , M. Feng , X. Liu , J. Yang , Fiber. Polym. 2021, 22, 1883–1888;

[2] (a) R. Chen , Y. Bin , Y. Nakano , N. Kurata , M. Matsuo , Colloid Polym. Sci. 2010, 288, 307–316;

[3] (a) N. Bahramian , M. Atai , M. R. Naimi-Jamal , Dental Materials 2015, 31, 1022–1029; 26113427 10.1016/j.dental.2015.05.011

[4] W. Li , L. Meng , R. Ma , Polym. Test. 2016, 55, 10–16.

[5] (a) X. Jin , W. Wang , C. Xiao , T. Lin , L. Bian , P. Hauser , Compos. Sci. Technol. 2016, 128, 169–175;

[6] Q. Gao , J. Hu , R. Li , Z. Xing , L. Xu , M. Wang , X. Guo , G. Wu , Radiat. Phys. Chem. 2016, 122, 1–8.

[7] M. Mohammadalipour , M. Masoomi , M. Ahmadi , S. Safi , RSC Adv. 2016, 6, 41793–41799.

[8] (a) R. Sa , Y. Yan , Z. Wei , L. Zhang , W. Wang , M. Tian , ACS Appl. Mater. Interfaces 2014, 6, 21730–21738; 25401775 10.1021/am507087p

[9] (a) Z. Qiao , X. Lv , S. He , S. Bai , X. Liu , L. Hou , J. He , D. Tong , R. Ruan , J. Zhang , J. Ding , H. Yang , Bioact. Mater. 2021, 6, 2829–2840; 33718665 10.1016/j.bioactmat.2021.01.039 PMC7905459

[10] M. Feng , W. Li , X. Liu , M. Huang , J. Yang , Polym. Test. 2021, 93, 106883.

[11] S. Chhetri , A. Sarwar , J. Steer , R. Dhib , H. Bougherara , Composites Part A 2022, 152, 106678.

[12] A. Kong , Y. Sun , M. Peng , H. Gu , Y. Fu , J. Zhang , W. Li , Colloids Surf., A 2021, 617, 126388.

